# Editorial: Effects of oral anticoagulant therapy in atrial fibrillation patients with comorbidities

**DOI:** 10.3389/fcvm.2023.1205870

**Published:** 2023-05-02

**Authors:** Runkai Li, Ping Yuan, Jianyong Ma, Wengen Zhu

**Affiliations:** ^1^ Department of Cardiology, The First Affiliated Hospital of Sun Yat-Sen University, Guangzhou, China; ^2^ Department of Cardiology, The Second Affiliated Hospital of Nanchang University, Nanchang, China; ^3^ Department of Pharmacology and Systems Physiology, University of Cincinnati College of Medicine, Cincinnati, OH, United States

**Keywords:** oral anticoagulant therapy, atrial fibrillation, comorbidities, bleeding, stroke

**Editorial on the Research Topic** Effects of oral anticoagulant therapy in atrial fibrillation patients with comorbidities

Atrial fibrillation (AF) is the most common arrhythmia in clinical practice and remains one of the primary contributors to cardiovascular diseases and mortality on a global scale. The incidence of AF increased exponentially with age, especially in the elderly with comorbidities such as hypertension, coronary artery atherosclerosis, valvular heart disease, acute or chronic kidney disease, or respiratory diseases. According to data from 2017, AF contributes to 0.51% of the burden of the global cumulative mortality. Moreover, the mortality associated with AF has increased by approximately 81% in the past 20 years (1). Among the complications of AF, stroke or systemic embolism (SSE) is the leading cause of morbidity and mortality, particularly with respect to cerebrovascular events. Studies have estimated that AF patients have a two-fold risk of stroke-related death compared to non-AF patients (2). Therefore, anticoagulant therapy for preventing thromboembolic events plays a crucial role in the long-term comprehensive management of AF patients, as it has important implications for improving patient prognosis, enhancing the quality of life, and extending overall survival (3).

As the cornerstone of anticoagulant therapy for AF patients, oral anticoagulants (OACs) significantly reduce the risk of thromboembolic events and all-cause mortality in AF patients (4). However, while anticoagulant therapy has clinical benefits, it may also be accompanied by corresponding bleeding side effects in clinical practice. Therefore, to establish the definite clinical benefits of OAC therapy in AF patients, several studies published in our research topic specifically focused on patients at a high risk of bleeding. The results revealed that in AF patients with a history of gastrointestinal (Zhao et al.) or intracranial hemorrhage (Liu et al.), OAC therapy can still reduce the risk of thromboembolic events, ischemic stroke, and all-cause mortality, and non-vitamin K antagonist oral anticoagulants (NOACs) exhibited more pronounced clinical benefits compared to vitamin K antagonists (VKAs). However, OAC therapy in AF patients with a history of intracranial bleeding also carries a higher risk of major bleeding. Furthermore, we also pay special attention to elderly AF patients, as this population has common characteristics such as impaired liver and kidney function, polypharmacy, multiple comorbidities, increased risk of falls and bleeding, a higher propensity for bleeding, and poor adherence to medication. As a result, selecting appropriate drugs and dosages for anticoagulant therapy in this population becomes crucial. To strike a balance between the risks of stroke-related mortality and anticoagulant-related bleeding in these patients, Zhao et al. developed and established a clinically applicable prediction model for the inappropriate use of NOACs based on a multicenter cohort of elderly AF patients. As the first prediction model for NOAC-related risks in elderly AF patients, it has important clinical value in evaluating anticoagulant-related bleeding risks in high-risk populations and optimizing anticoagulant therapy for AF patients.

In order to reduce the risk of bleeding in AF patients undergoing anticoagulant therapy, the precise assessment of bleeding event risks has been a focal point of clinical research. Currently, Liu et al. have conducted a meta-analysis to further clarify the comparative accuracy of prediction, suggesting that the ORBIT score did not show a significant advantage over the classic HAS-BLED score in terms of predicting bleeding risks. In addition, regular monitoring of coagulation function is crucial for AF patients on long-term anticoagulant therapy, particularly those using warfarin. However, in certain situations that require urgent surgical or interventional procedures, routine laboratory tests are not suitable for promptly assessing coagulation function. Viscoelastic tests can rapidly provide information on residual levels of NOACs in plasma, which has significant value in understanding patients' coagulation function status, formulating specific surgical plans, preparing preoperative measures, and assessing surgical risks in emergency care. However, there is still controversy regarding the accuracy of measuring residual NOAC plasma concentrations with viscoelastic tests. Therefore, Sahli1 et al. conducted a meta-analysis of relevant articles on viscoelastic tests and the results indicated that viscoelastic tests still have important value in providing real-time information about residual NOAC activity, although the sensitivity for quantifying residual NOAC concentrations in plasma needed to be improved.

In the selection of oral anticoagulants, NOACs have gained widespread attention since their introduction. Compared to VKAs, NOACs selectively inhibit the activity of a single molecular target, either thrombin or factor Xa, and do not require monitoring of coagulation function or frequent dose adjustments. NOACs are also not restricted by interactions with other foods or drugs, offering a stable anticoagulant effect and a favorable safety profile with lower bleeding risk (5). However, when anticoagulant therapy is required for AF patients with concurrent comorbidities such as cardiovascular, pulmonary or renal diseases, some factors may complicate the situation, such as changes in drug metabolism and clearance, the necessity of drug dose adjustments, increased bleeding risk, and consideration of alternative treatment options. Therefore, Ren et al. previously investigated the effectiveness and safety of NOACs in subpopulations of AF patients with different comorbidities. Their results showed that compared to VKAs, the use of NOACs in populations at risk of kidney disease can reduce the risk of acute kidney injury in AF patients. In addition, Li et al. found that in AF patients with concomitant end-stage renal disease on dialysis, the use of NOACs showed at least similar effectiveness and safety results compared to VKAs. For patients with concomitant pulmonary diseases, the effects of VKAs and NOACs on reducing the risk of recurrent thromboembolic events were not significantly different statistically, but oral anticoagulant therapy significantly reduced the risk of death in AF patients with concomitant pulmonary hypertension, pulmonary embolism, and chronic obstructive pulmonary disease (Lai et al.). Furthermore, several authors also analyzed the use of anticoagulant therapy in AF patients with cardiovascular diseases such as myocardial infarction and heart failure, suggesting that NOACs were superior to warfarin in stroke prevention (Yu et al. Lee et al. Wulamiding et al.).

In conclusion, while emphasizing the importance of anticoagulation therapy in patients with AF, it is also crucial to evaluate the bleeding risk of individual patients. Oral anticoagulant therapy has demonstrated significant benefits in preventing ischemic stroke and reducing mortality in AF patients with comorbidities. A series of clinical studies have confirmed the effectiveness and safety of oral anticoagulant therapy, especially NOACs, in patients with AF. Similarly, for individual patients, a personalized anticoagulation management plan needs to be established based on the assessment of the bleeding risks. However, the safety of NOACs in certain populations, such as AF patients with concomitant mitral stenosis or aortic stenosis, pulmonary arterial hypertension or pulmonary fibrosis, and severe renal insufficiency, remains controversial, which requires further well-designed trials with a larger sample size to validate previous findings.
